# Correlation Network Analysis reveals a sequential reorganization of metabolic and transcriptional states during germination and gene-metabolite relationships in developing seedlings of Arabidopsis

**DOI:** 10.1186/1752-0509-4-62

**Published:** 2010-05-13

**Authors:** Elizabeth Allen, Annick Moing, Timothy MD Ebbels, Mickaël Maucourt, A Deri Tomos, Dominique Rolin, Mark A Hooks

**Affiliations:** 1 School of Biological Sciences, Bangor University, Bangor, Gwynedd LL57 2UW, UK; 2 INRA, Université de Bordeaux, UMR619 Fruit Biology Unit, BP 81, F-33140 Villenave d Ornon, France; 3 Division of Surgery, Oncology, Reproductive Biology and Anaesthetics, Sir Alexander Fleming Building, Imperial College London, London SW7 2AZ, UK; 4 Plateforme Métabolome du Centre de Génomique Fonctionnelle Bordeaux, IFR103 BVI, BP 81, F-33140 Villenave d'Ornon, France

## Abstract

**Background:**

Holistic profiling and systems biology studies of nutrient availability are providing more and more insight into the mechanisms by which gene expression responds to diverse nutrients and metabolites. Less is known about the mechanisms by which gene expression is affected by endogenous metabolites, which can change dramatically during development. Multivariate statistics and correlation network analysis approaches were applied to non-targeted profiling data to investigate transcriptional and metabolic states and to identify metabolites potentially influencing gene expression during the heterotrophic to autotrophic transition of seedling establishment.

**Results:**

Microarray-based transcript profiles were obtained from extracts of Arabidopsis seeds or seedlings harvested from imbibition to eight days-old. ^1^H-NMR metabolite profiles were obtained for corresponding samples. Analysis of transcript data revealed high differential gene expression through seedling emergence followed by a period of less change. Differential gene expression increased gradually to day 8, and showed two days, 5 and 7, with a very high proportion of up-regulated genes, including transcription factor/signaling genes. Network cartography using spring embedding revealed two primary clusters of highly correlated metabolites, which appear to reflect temporally distinct metabolic states. Principle Component Analyses of both sets of profiling data produced a chronological spread of time points, which would be expected of a developmental series. The network cartography of the transcript data produced two distinct clusters comprising days 0 to 2 and days 3 to 8, whereas the corresponding analysis of metabolite data revealed a shift of day 2 into the day 3 to 8 group. A metabolite and transcript pair-wise correlation analysis encompassing all time points gave a set of 237 highly significant correlations. Of 129 genes correlated to sucrose, 44 of them were known to be sucrose responsive including a number of transcription factors.

**Conclusions:**

Microarray analysis during germination and establishment revealed major transitions in transcriptional activity at time points potentially associated with developmental transitions. Network cartography using spring-embedding indicate that a shift in the state of nutritionally important metabolites precedes a major shift in the transcriptional state going from germination to seedling emergence. Pair-wise linear correlations of transcript and metabolite levels identified many genes known to be influenced by metabolites, and provided other targets to investigate metabolite regulation of gene expression during seedling establishment.

## Background

Germination is a phenomenon with complex regulation that is a balance between the release of dormancy and the promotion of germination. This reflects the relationship between the hormones gibberellic acid (GA) and abscisic acid (ABA), environmental cues [1,2], and the spatial distribution of hormone action and gene expression [3-5]. Considerable effort has been put into elucidating the molecular mechanisms controlling seed germination with greater application of gene expression profiling. These studies have highlighted the roles of gene expression changes in mediating GA and ABA interactions in the control of dormancy and germination [6-10]. To complement the growing number of gene expression studies, Fait *et al*. [11] conducted an integrated metabolomic and gene expression study of various seed developmental stages from maturation through germination. They identified distinct metabolite profiles associated with the various developmental stages and suggested that seeds are metabolically primed for germination during desiccation and subsequent metabolic programming during imbibition and germination is essential for seedling establishment. An integrated metabolomic and transcriptomic study of photomorphogenesis in red light and far-red light treated seedlings showed that even though transcript profiles were relatively similar, phenotypic differences could be explained by significant differences at the level of the metabolome [12].

Prior to seed germination, the mobilization of stored triacylglycerol (TAG) begins in earnest in order to feed the developing seedling. The processes by which germination and lipid mobilization are regulated have been found to be distinct [13], and it is likely that reserve mobilization is governed by abscisic acid-related processes within the embryo [4]. In Arabidopsis, stored sugars are consumed by the time the radicle has emerged, and within 48 h after germination lipid and protein stores have been consumed [14]. At this point, the seedling must become photosynthetically competent. It has been suggested that metabolic signals may regulate the transition from heterotrophy to autotrophy in seedlings in order to maximize the use of storage compounds [15]. Exploiting the altered behavior of seed germination and of seedling vigor for forward genetic screens of Arabidopsis mutants has been instrumental in revealing the potential signaling properties of metabolites, primarily sugars [16], and nutrients [17]. Mutant studies have revealed the interaction of sugars and hormones [18,19] and the concept of a carbon:nitrogen 'matrix effect' in metabolic regulation [20]. Through a forward genetic screen using the toxic analogue monofluoroacetic acid, we identified mutants disrupted in their ability to metabolize exogenously supplied acetate through the glyoxylate cycle [21,22]. A physiological analysis of the mutants provided evidence that carbohydrate responses of seedlings may be impaired within the mutants. This suggests a cross-talk between organic acid and carbohydrate signaling in developing seedlings [22] with the possibility of either acetate or down-stream metabolites influencing gene expression in developing seedlings.

Many forward genetic screens have relied on observing differential sensitivity of mutants to added compounds. This approach does not work for many metabolites, since artificially high concentrations must be used and undesired traits are selected. For example, organic acids pose this problem because they are weak acids, and mutant selection for specific responses may be confounded by responses to altered intracellular pH. Integrated analysis of metabolite and transcript data offers a way to identify co-regulatory networks of metabolites and genes [23,24]. This has been applied successfully to identify potential genetic regulation of metabolite levels concerning sulfur stress [25-29], glucosinolate metabolism [30], and nitrogen responses [31,32] in Arabidopsis and fruit development in tomato [28,33-35]. The suggestion that strong correlations between metabolites and transcripts may reflect metabolite effects on gene expression [27,28,36], therefore, enables integrated analysis to be used to identify potential signaling metabolites for subsequent detailed studies. We obtained metabolite and transcript profiling data from a series of samples spanning germination and establishment, and analyzed the data to identify pair-wise combinations of genes and metabolites strongly correlated over this developmental transition. We discuss how analysis of metabolite-gene correlations provided evidence for differential regulation of a common ontological class of genes. Furthermore, the network correlation analysis approach can provide supplemental information on the progression of metabolic and transcriptional states during developmental transitions [27,28]. Both types of profiling data were mined for interesting gene expression and metabolite patterns and relationships. Principle Component Analysis (PCA) and network correlation analysis based on spring-embedding [37] were used to integrate and visualize the data to obtain information about the metabolic and transitional states present during germination and seedling development.

## Results

### Gene expression during seedling development

**Figure 1:**
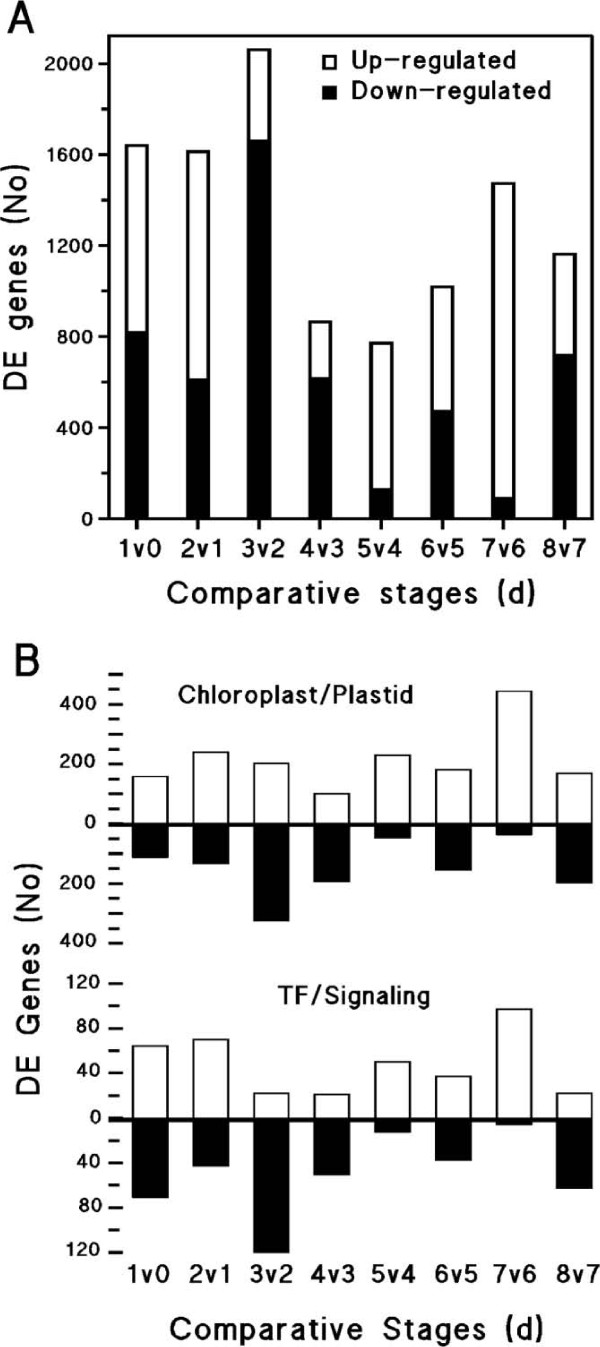
**Trends in differential gene expression**. DE genes were determined between each successive day at a threshold cut-off level of 1.5-fold. Each comparative stage, i.e. day, was measured in triplicate and the mean of the hybridization intensities calculated prior to DE analysis. (A) Total number of DE genes and the split between UR and DR genes. (B) The proportion given as percentages of total DE genes comprised by either chloroplast/plastid protein or TF/signaling protein encoding genes as given in the TAIR gene ontology database. Open and closed bars represent UR and DR genes, respectively.

The combined use of a threshold cut-off value of 1.5-fold and 99% confidence limits for statistical significance produced 10,605 differentially-expressed (DE) genes in total, both up-(UR) and down-regulated (DR) over the eight pairwise comparisons (Fig. 1A). This total number of DE events is similar to those reported in analogous studies. For example, over 10 stages of development of tomato exposed to ethylene, Alba *et al*. [38] estimated that almost 3,500 DE events would have occurred. They concluded that this was a large underestimate for the fruit as a whole, since only the pericarp was analyzed. Between days 0 and 1 and days 1 and 2 there appear to be an equivalent total number of DE genes divided equally between those UR and DR. Between days 2 and 3, there is an increase in DE genes of about 25%. Notably, DR genes comprised more than 80% of the DE genes between these days. There was a 2-fold decrease in total DE genes between days 3 and 4, which was a majority of DR genes. The number of DE genes begins to increase in later days, but with UR becoming more predominant at alternate stages. This was most apparent comparing days 4 and 5 and days 6 and 7. Using the ontological assignments available at TAIR, we looked more closely at two different classes of genes, nuclear genes encoding chloroplast and plastid proteins and those encoding transcription factors (TF) and signaling genes (Fig. 1B). The former would indicate changes to the autotrophic state, whereas the latter would reflect overall regulatory activity. In general, the expression profiles of these classes of genes were similar, which is not unexpected with the requirement for transcriptional control of photosynthetic development. The differences in the number of DE genes observed between the two classes preceded emergence, which occurred from day 2 to day 3. From day 2 to day 3 other ontological classes associated with regulatory processes, such as nucleic acid binding or kinase activity were proportionately higher among DR genes compared to UR genes (data not shown). There was substantial UR of both TF/signaling and chloroplast/plastid gene expression at days 5 and 7 when compared with the previous day. The proportions of each ontological class among both UR and DR genes were similar at day 5 compared with day 4, with only cell wall-classified genes showing a relative higher proportion the UR category (3% UR *versus *0.8% DR, data not shown). This was also the case for day 7 compared with day 6 with only the receptor binding class appearing substantially DR (0.8% *versus *0.1%). These are processes that are occurring primarily in cotyledons and the hypocotyl leading to leaf growth, since true leaves do not make up a substantial proportion of seedling mass until about day 8 [39].

### Behavior of metabolites during development

A total of 27 metabolites corresponding to a variety of known and unknown metabolites including four soluble carbohydrates, nine amino acids and five organic acids were quantified from the ^1^H-NMR spectra of seedling extracts. Although these metabolites comprise a small proportion of the total metabolic complement of a cell, these metabolites are the most abundant ones. They reflect the nutritional state of the tissue as an immediate source of carbon and/or nitrogen and serve as respiratory substrates for energy production. A direct comparison of data from our NMR profiling platform with GC-MS acquired data demonstrated a similar capacity to distinguish metabolic states [40]. Additionally, a number of the metabolites are well known effectors of gene expression and some, such as sucrose, isoleucine and glutamine, have high regulatory potential as determined by correlation analysis [28]. The values obtained for each metabolite are given in Additional File 1.

**Figure 2:**
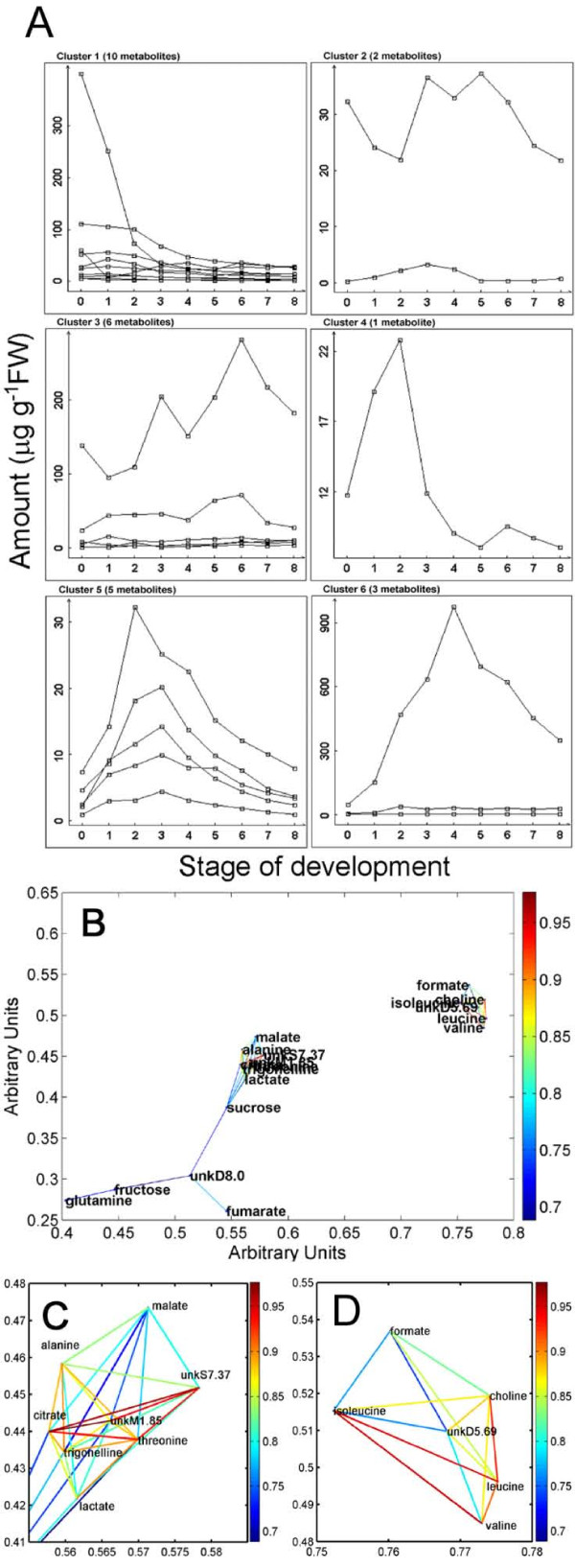
**Relationships between metabolites**. (A) Clusters of metabolites with similar profiles generated by 2D-SOM. Hierarchal and *K*-means clustering were used to estimate the optimal number of bins for 2D-SOM analysis. Metabolites in cluster 1: sucrose, rhamnose, citrate, alanine, trigonelline, lactate, glucose, threonine, unkS7.37, unkM1.85; Cluster 2: arginine, formate; Cluster 3: fumarate, proline, glutamate, unkD8.0, unkM5.18, unkD3.12; Cluster 4: malate; Cluster 5: valine, isoleucine, leucine, choline, unkD5.69; Cluster 6: fructose, glutamine, unkM7.9. (B) Spring embedding plots showing relationships based on correlations. The plot shows metabolites as nodes and Pearson correlation coefficients over days as connections. The color of the connecting line describes the strength of the correlation between the nodes; a dark red color indicates a strong positive correlation and a dark blue line represents a weaker positive correlation according to the scale of correlation coefficients on the right of the graph. Only correlations above a Bonferroni-adjusted P-value < 0.0001 are shown. (C) Enlargement of the lactate cluster. (D) Enlargement of the valine cluster. Since values start from an initial random configuration, the directions separating cluster in each spring embedding plot are arbitrary, but they provide an indication of distance separating nodes and edges.

In order to understand better the general trends in metabolite behavior over the developmental series we produced two-dimensional self organising maps (2D-SOMs) that grouped like-varying metabolites (Fig. 2A). A number of metabolites decreased throughout the developmental period shown in Cluster 1. As expected, this cluster included sucrose [13,41], which is known to decrease upon the initiation of germination. Clusters 2 and 3 contain metabolites that fluctuate with no particular trend or increase slightly during development. Clusters 4-6 contain 9 metabolites that show a biphasic profile of increasing then decreasing levels. Malate (cluster 4) shows a relatively sharp increase and decrease compared to valine, leucine and isoleucine (cluster 5) although each attains a maximum level on the same day. Cluster 6 shows 3 metabolites, glutamine, fructose and an unknown compound that attain maximum levels about a day later. We are particularly interested in metabolites that changed over the course of development (or part of it), since they would be candidates for metabolic control factors.

The relationships between individual metabolites are clearer when correlations are included in the visualization as shown in the spring embedding plots (Fig. 2B-C). Our threshold *p*-value produced a correlation coefficient cut-off value of 0.68. We observed significant correlations between 19 metabolites that appear to be separated into three clusters. The cluster containing glutamine, fructose, fumarate and unkD8.0 are linked to the lactate cluster (Fig. 2B) via sucrose. The lactate cluster contains those metabolites that are decreasing over time, such as trigonelline, threonine, citrate, and alanine (Fig. 2C). Malate has also been included within this cluster and has a relatively high correlation to alanine. This could reflect a partitioning of malate into alanine either via oxaloacetate or pyruvate. The third cluster (Fig. 2D) consists of the aliphatic amino acids and the compounds choline, and formate. It was expected that the three amino acids leucine, isoleucine and valine would be highly correlated as they share common synthetic and catabolic pathways.

### Transcriptional states of developing seedlings

**Figure 3:**
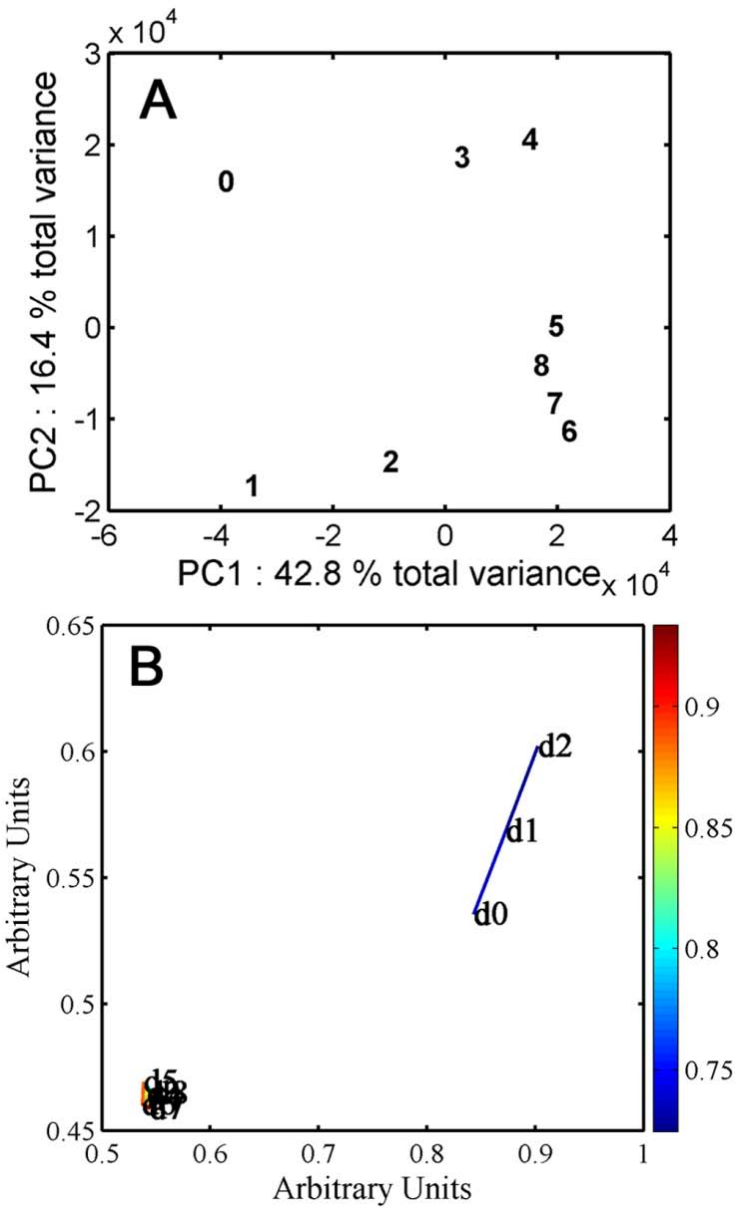
**Day-by-transcript relationships**. (A) PCA scores plot of the time points sampled during germination and seedling establishment based on the average transcript levels. Each number 0 to 8 represents one day (24 h) from imbibed seeds (0) to 8 days (8) of age, respectively. (B) Higher order relationships among days based on mean values of transcript levels from the 3 replicates visualized by spring embedding. The plot shows day 0 (d0) to day 8 (d8) as nodes and the relative degree of transcript correlation as edges. Clustering was based on Pearson correlation coefficients at a threshold cut-off of 0.7. The color bar on the right of the figure provides the relative degree of correlation.

In order for us to compare transcriptional states among days, only genes that were expressed at each of the 9 sampling points were included. However, in order to maximize the number of genes in the analysis only one expression value per time point was required. This filtering process resulted in a final set of 10,235 genes (Additional File 2). Initially, a principal component analysis (PCA) scores plot was produced in order to investigate the relationships among days according to gene expression profiles (Fig. 3A). This revealed a general progression of time points across PC1 with the transition from day 2 to 3 and from day 4 to 5 contributing mostly to PC2. Days 5 to 8 appear to form a loose cluster, which would be expected if the expression of photosynthetic genes has begun in earnest by day 5, which agrees with the gene expression profiling data (Fig. 1). The PCA scores plot for individual sample is given in Additional File 1. The loadings analysis indicated that the most variant genes were chlorophyll a/b binding proteins and small subunits of ribulose bisphosphate carboxylase (data not shown). It is also evident that PC2 comprises some technical variation due to differences in slide hybridization since the apparent outliers do not correspond to any one particular sampling set. Spring embedding was used to investigate further the relationships between time points in the dataset of transcript profiles [37]. The spring embedding algorithm is non-linear, and so is able to amplify any clustering in the data to make it more visually clear compared to standard PCA analysis. Due to the size of the data set and the possible number of correlations that can be obtained, the cut-off threshold was set to 0.7. The spring embedding was clearer in showing the division of tissue samples into two clusters comprising days 0-2 and days 3-8 (Fig. 3B). When the threshold was dropped to 0.6 the connections between day 2 and the later days became more apparent and the spring embedding plot began to mimic the PCA plot with day 2 moving from day 1 and lying more closely to days 6 to 8 than to day 3.

### Metabolic states of developing seedlings

**Figure 4:**
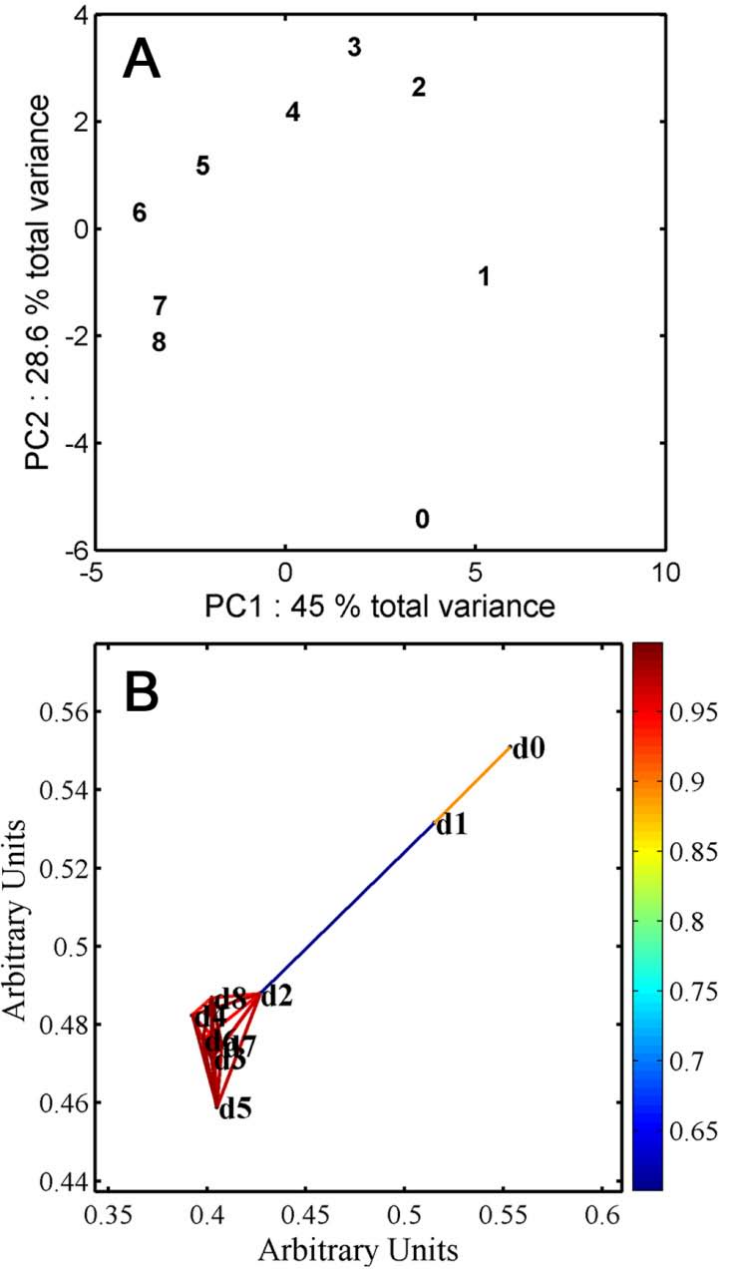
**Day-by-metabolite relationships**. (A) PCA scores plot where each number represents one day (24 h) from imbibed seeds (0) to 8 days of age (8). (B) Spring embedding plot where the symbols d0 to d8 correspond to the samples in A. Each point is a node representing the mean value and each line gives the relative degree of correlation. The threshold Pearson correlation coefficient for the spring embedding was 0.7. The color bar on the right of each figure provides the relative degree of coloration. Both types of analysis were based on the mean values (n = 3) of 3 replicates (2 replicates for day 3).

A PCA scores plot of time points based on metabolite profiles revealed a curvature in the points (Fig. 4A). Variation among days 0 and 2 was shown almost exclusively in PC2 and subsequent differences to day 8 were shown in gradual shifts in both PC1 and PC2. The PCA scores plot for individual samples is given in Additional File 1. The loading plots confirmed our conclusions from the visual inspection of the data in that the major differences between days 0 and 1 were the levels of metabolites that decreased substantially, such as sucrose, glucose and unkM5.18 (Additional File 1). The clustering in the PCA loadings plot mirrored that of the spring embedding plot for the metabolites alone (Fig. 2) and suggested a steady transition in states from day 1 to later days. Spring embedding was used to clarify the relationships among the days, based on the metabolite data (Fig. 4B). At a threshold correlation value of 0.6 two clusters became apparent. There was a relatively high correlation between day 0 and day 1 and among days 2-8, with a lower correlation between day 1 and day 2. As the threshold correlation is decreased the groups move closer together, but the clustering was not lost until a correlation cut-off below 0.5 was used. If the threshold cut-off is increased to 0.7, then the link between day 1 and day 2 is severed. The apparent separation of day 0 from day 1 is due mainly to the second replicate sample of day 1 (Additional File 1). The other two day 1 samples clustered very closely to the three day 0 samples and all the samples from day 2 to day 8 showed a strong correlation. In order to identify the metabolites with a significant difference in measured levels between days 1 and 2, which are the developmental stages that mark the division of the two clusters, we applied a Student s t-test to the data (FDR < 0.1). A significant difference was observed for the levels of several metabolites (Additional File 1). We can only speculate that relatively high sucrose, rhamnose, lactate, citrate, alanine, trigonelline and unkM1.85, and relatively low fructose, glutamine and unkD8.0 comprise part a metabolic state that is conducive for germination, and that change in these metabolites promotes emergence and establishment. Less abundant metabolites that were not quantifiable by NMR also will be very important in defining metabolic states.

### Metabolite and transcript co-analysis

**Figure 5:**
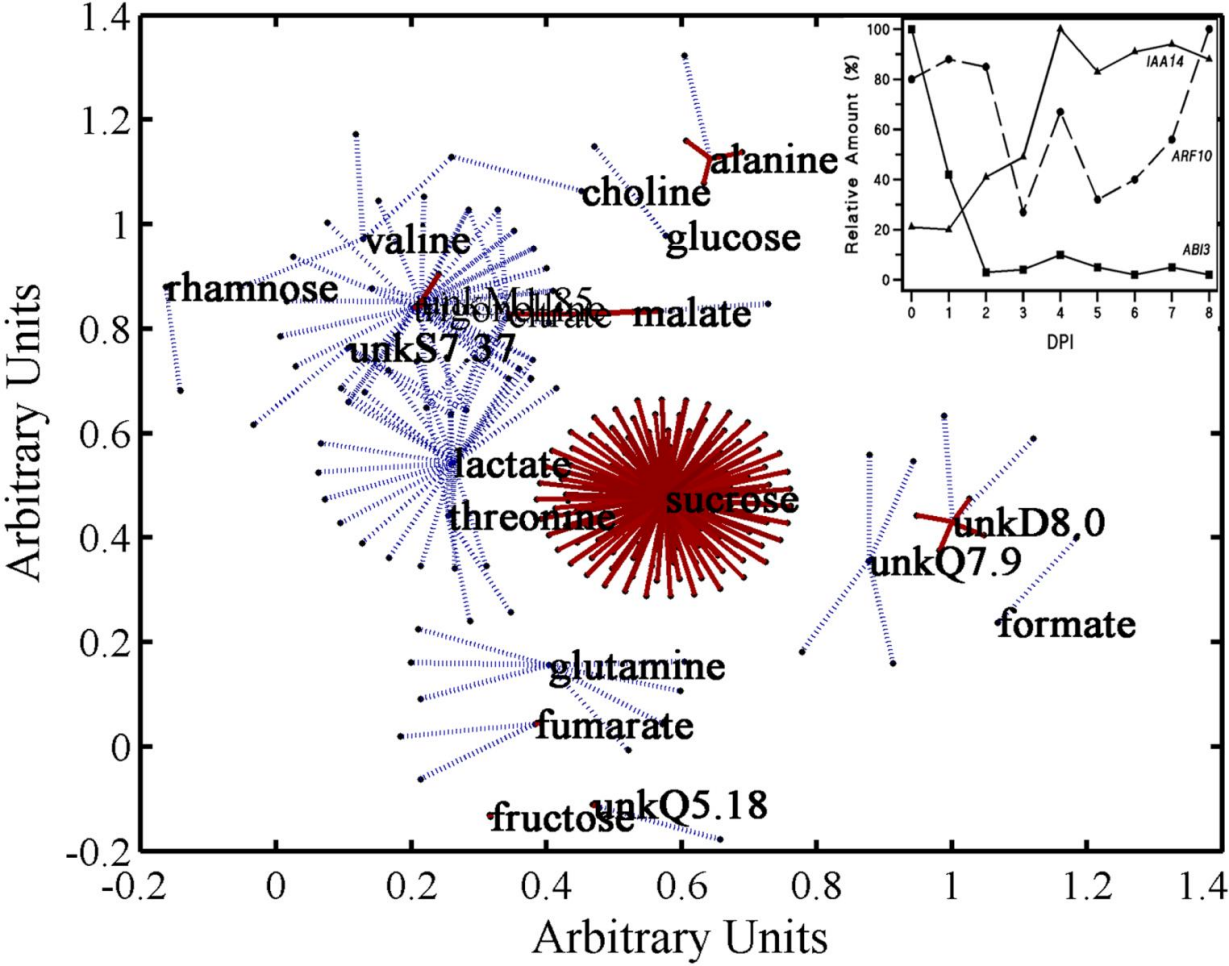
**A spring embedding model revealing relationships between metabolites and genes from days 0 to 8**. Pearson correlation coefficients were determined between every metabolite and gene over the 9 time points. Metabolites are central nodes from which connected genes radiate outwards. The coloured lines represent edges describing the nature of the correlation; a dark red line represents a strong positive correlation whereas a dark blue line represents a strong negative correlation. A total of 237 correlations were identified between 20 metabolites and 209 genes at the threshold cut-off of (p < 0.0001, r > |0.95|). The plots inset show the profiles of the average expression values for the transcription factors *IAA14*, *ARF10 *and *ABI3 *used to calculate correlation coefficients.

The majority of the metabolites measured demonstrated altered levels throughout the time course allowing correlations to be identified with gene transcript levels. Spring embedding was used to visualize relationships between genes and metabolites based on significant correlations over all the sampling points (Fig. 5). Using a false discovery rate (FDR) [42] of 10% to generate the threshold P-value (see legend), a total of 237 pair-wise correlations were identified among 20 metabolites and 210 genes (Additional File 3). We emphasize that the Bonferroni and Benjamini and Hochberg FDR adjustments that were used to establish thresholds of significance are very stringent. Nevertheless, in order to check our use of a FDR threshold, the time point labels of the gene and metabolite data were randomly permuted 1000 times and each time the cross-correlations were calculated using the same threshold level of significance Across the 1000 permutations, the median number of significant correlations was 28. This corresponds to 11% of the 237 seen in the non-permuted data and is close to the desired FDR of 10%.

The metabolites are presented as nodes to which the correlated genes radiate outwards. As expected, both positive and negative correlations were identified. Table 1 lists the metabolites identified as showing a correlation with one or more genes along with the nature of the correlation(s). The metabolite profile is described as increasing, decreasing, or as biphasic throughout the developmental series. The gene ontology from the TAIR database was used to identify the function for each gene listed. Of the 237 correlated genes, 19% were identified in the TAIR database as encoding an "expressed" or a "hypothetical" protein. Of the remaining 196 correlations, 25% of the genes were associated with a known regulatory aspect of plant development, for example, phytohormone or light response, or had previously been identified as demonstrating seed-specific expression. A further 12% of the genes with an assigned identity in the TAIR database were involved with signal transduction. Sixty-one percent of the genes (129 out of 210) showed significant correlation with sucrose. All sucrose-gene correlations were positive, since the FDR only gave the most significant correlations, which were independent of sign. Most of the regulatory/signal transduction genes correlated with sucrose indicating that they decrease rapidly upon transfer of seeds to germination conditions. These included the transcription factors *PHYTOCHROME INTERACTING FACTOR 1 *(*PIF1*), *ABSCISIC ACID INSENSITIVE 3 *(*ABI3*), and *ATMYB56 *(AT5G17800), and the light-receptor/kinase genes *PHYA *and *PHYD *(Table 2). Correlations with other metabolites revealed progressive changes in the transcript level of other transcription factors that might interact, such as *Auxin Response Factor 10 *(*ARF10*) and *ABI3 *[43] and the Aux/IAA protein family TF *SOLITARY-ROOT *(*SLR*)/*IAA14*. As *ABI3 *drops immediately following transfer to growth conditions, *ARF10 *remains constant and does not drop until after germination, and *IAA14 *is present in imbibed seeds and then increases prior to germination (Fig. 5, inset).

**Table 1:** Correlations between metabolites and transcript levels in developing seedlings.

Metabolite	Number of Relationships*	Direction of Correlations†
Glucose	1	N
Sucrose	129	P
Fructose	1	P
Rhamnose	1	N
Malate	3	N (1), P (2)
Citrate	7	N (6), P (1)
Fumarate	3	N (2), P (1)
Formate	1	N
Lactate	21	N
Glutamine	7	N
Alanine	4	N (1), P (3)
Valine	3	N
Threonine	5	N
Trigonelline	16	N (15), P (1)
Choline	1	N
unkD8.0	6	N (2), P (4)
unkM7.9	4	N
unkS7.36	3	N
unkM5.18	2	N (1), P (1)
unkM1.85	19	N (18), P (1)

*Number of significant Pearson correlations between the metabolite and genes above *p *= 9.8 × 10^-5 ^(FDR = 5%) determined over the 9 sampling periods, day 0 to day 8. †The direction of the linear regression, either positive (P) or negative (N) and the number in each direction in parentheses.

**Table 2:** Identified regulatory genes correlated with metabolites.

Locus	Metabolite	*r *= †	Gene Symbol	Gene Description†
*Transcription factors*				
AT1G30970	Sucrose	0.9672	SUF4	Suppressor of Frigida 4
AT2G20180	Sucrose	0.9493	PIF1	Myc-related bHLH transcription factor
AT2G24500	Sucrose	0.9628	FZF	C2H2 zinc finger protein FZF
AT2G25930	unkM7.9	-0.9752	ELF3	Early Flowering 3
AT2G28350	unkM7.9	-0.9667	ARF10	Auxin Response Factor 10
AT2G31370	fumarate -	0.9495	POSF21	bZIP transcription factor POSF21
AT2G43010	unkM1.85	-0.9529	PIF4	Nuclear localized bHLH protein that interacts with active PhyB
AT3G15030	unkS7.37	-0.9586	TCP4	Arabidopsis thaliana TCP Family transcription factor
AT3G24650	Sucrose	0.9721	ABI3	Homologous to the maize transcription factor Viviparous-1.
AT4G14550	unkM1.85	-0.9588	SLR/IAA14	IAA14 is a member of the Aux/IAA protein family. Solitary Root locus.
AT4G35570	Sucrose	0.9803	HMGB5	High Mobility Group B 5
AT5G17800	Sucrose	0.977	ATMYB56	Member of the R2R3 factor gene family
				
*Kinases *&*Receptors*				
AT1G09570	Sucrose	0.9777	PHYA	Phytochrome A
AT4G16250	Sucrose	0.9581	PHYD	Phytochrome D
AT3G16030	alanine -	0.9519	CES101	Callus Expression of RBCS
				
*DNA/RNA binding*				
AT1G61040	Sucrose	0.9495	VIP5	Vernalization Independence 5 putative heterogeneous nuclear ribonucleoprotein
AT2G33410	Sucrose	0.9771		
AT2G37020	Sucrose	0.9522		DNA binding, Chloroplast
AT3G16810	Sucrose	0.9735	APUM24	Arabidopsis Pumilio 24
AT4G14520	Sucrose	0.9625		Homologous to the DNA-directed RNA polymerase II subunit (At5g59180)
AT4G25500	Sucrose	0.9721	ATRSP35	arginine/serine-rich splicing factor
AT4G36020	Sucrose	0.9577	CSDP1	Cold shock domain protein
AT5G07290	Sucrose	0.9538	AML4	ARABIDOPSIS MEI2-LIKE 4
AT5G14270	glutamine	-0.9566	ATBET9	Arabidopsis thaliana Bromodomain and Extra terminal Domain protein 9
AT5G38890	Sucrose	0.9898		Exoribonuclease-related
AT5G53180	fumarate -	0.9593	ATPTB2	Polypyrimidine tract-binding (PTB) Protein

† Pearson correlation coefficients based on a false discovery rate at a significance threshold < 0.05. The sign gives the direction of correlation.

An example of the relationships between a metabolite and a connected gene is given for lactate (Additional File 1). Lactate was negatively correlated with 21 genes, five of which had no assigned identity in the TAIR database. Of the 17 genes with an assigned identity, seven showed an involvement in photosynthetic-related functions. Eight of the genes within the group are also affected by abiotic or biotic stress. These include 1-aminocyclopropane-1-carboxylate oxidate (ACO4, At1g05010), calmodulin-like 9 (At3g51920) and the PIP2A aquaporin (At3g53420), which is induced during dehydration stress. Due to our stringent statistical cut-off, none of the 21 genes of the lactate cluster include any of the 19 hypoxia inducible genes reported by Loreti [44]. However, the genes encoding alanine aminotransferase (At1g17290), alcohol dehydrogenase (At1g77120), Class I non-symbiotic hemoglobin (At2g16060), and pyruvate decarboxylase 1 (At4g33070) reported as anoxia inducible by Sachs *et al*. [45] - and which are included in the set of 19 inducible genes - were positively correlated with lactate at a *p*-value less than 0.03 (r > 0.72). Although a number of gene expression profiles were correlated with more than one metabolite concentration, it was observed that of the seven photosynthetic genes correlated to lactate, only three showed correlations with other metabolites. Besides the two TFs shown in Table 2, the unknown metabolite unkM1.85 was negatively correlated with 17 other genes, seven of which were assigned photosynthetic functions.

## Discussion

More and more, integrative approaches are being employed to describe the function of molecular systems in development (for reviews see [24,46-49]. Whereas most metabolite-gene interaction studies have been from the point of view of understanding the genetic bases for changes in metabolism, such studies can be integral to understanding the control of gene expression by metabolic factors [28,49]. In fact, strong correlations between metabolite and transcript levels more likely reflect metabolite regulation of transcription than *vice versa *[36]. A recent study reported that a series of distinct metabolic switches were characteristic of the transition from dormant, dry seed to germinating embryo [11]. The results presented in this work extend the analysis to provide an overview of metabolite and transcriptome profiles from imbibed, non-dormant seeds through to established seedlings with the aim of identifying potential targets of metabolic control during seedling development covering the heterotrophic to autotrophic transition.

## Differential gene expression activity coincides with developmental stage

In the present work, we observed a relatively high level of transcriptional change occurring over the first three days of seedling development, which encompasses germination and emergence. Fifty percent of all DE events occurred during the first three days. Of these, more than 7% were genes categorized as either TF or signaling genes. This implies large changes in transcriptional activity during emergence. Alba *et al*. [38] made a similar conclusion from their analysis of gene expression in developing pericarp of ethylene treated tomatoes. Of 628 known DE genes during the 10 developmental stages they analyzed, 11% were either TF or signaling genes. We observed a substantial increase in DE at day 3 compared to day 2, with most genes comprising both chloroplast/plastid and TF/signaling genes. A high degree of transcriptional alteration may not be required for seedling development (i.e. cell division or differentiation) at this time, since they are geared for a constant rate of lipid degradation [50,51], and cell expansion is the principal means of seedling growth [14]. Subsequently, relative gene UR and DR appears to follow a cyclic pattern during the subsequent days, and it is interesting that this corresponds to likely transitional stages of development. Transcriptional transition is lowest between days 2 and day 4, since TF gene expression is DR. By day 4, all lipid reserves have been depleted and so by day 5 any potential catabolite repression would be eliminated to permit full development of autotrophy, which would be revealed by a relative increase in UR genes, such as those encoding chloroplast/photosynthetic proteins. Between days 6 and 7 rapid leaf growth begins [39] and a corresponding UR of gene expression may ensue. Accordingly, spikes of chloroplast/plastid and TF/signaling UR take place at days 5 and 7.

### Metabolic state establishes prior to germination and the switch in transcriptional programming

The changes to levels of various metabolites going from imbibition to early germination follow similar patterns as reported previously [11,13]. Fait *et al*. [11] observed a change of metabolic activity during post-imbibition germination 24 h after transfer of seedlings from cold to a germination inductive temperature. The grouping of the metabolite profiles produced during this experiment supports these findings, demonstrating that in the cold, a relatively stable metabolic state for the major metabolites is present and then changes relatively little for 24 h. A larger metabolic switch occurs from day 1 to day 2 but the metabolic state stabilizes during seedling establishment even though a number of metabolites show transient increases (Fig. 4).

Although gene expression profiles are changing from day 0 to day 2, there is a more dramatic change from day 2 to day 3 (Fig. 3). By this point, the seedling has emerged, but attainment of full photosynthetic competence does not appear to happen quickly. From the large DR of expression from day 3 to day 4, it is interesting to speculate that prior to the emergence of the radicle, *i.e*. by day 2, the embryo has attained a metabolic state that primes the seedling to reduce aspects of gene expression in preference for emergence and reserve mobilization.

### Revealing potential metabolic signals by correlation analysis

Deciphering metabolic contributions to switches in transcriptional states, such as observed during seedling development, will entail identifying individual signaling metabolites, the genes they affect and the concerted degree of affect [52]. Although any metabolic regulation during the heterotrophic to autotrophic transition would be complex, it should be possible to identify metabolites involved in signaling gene expression events by examining their behavior in relation to the expression of specific genes [36]. We determined linear correlations between each metabolite-gene pair with the assumption that the strength of correlation would indicate the potential for a regulatory relationship to exist (Fig. 5). Sucrose levels showed positive correlations with 129 gene transcripts. Comparison of the gene transcripts correlated with sucrose levels with previous microarray experiments and online databases showed that 44 of the 129 genes had previously been identified as sucrose-responsive [36,53-56]. The correlation of sucrose levels with a large proportion of previously identified sucrose-responsive gene transcripts reinforces the validity of the use of correlation coefficients to identify interesting relationships. Two well-studied TFs that were highly correlated with sucrose were *PIF1 *and *ABI3*. PIF1 has been identified as a negative regulator of photomorphogenesis in seedlings [57,58] and ABI3 may play a role in sugar-induced seedling developmental arrest [59]. The TF genes *IAA14 *and *ARF10 *showed high negative correlation with the unknown metabolites unkM1.85 and unkM7.9, respectively. In order for correlations to be identified a gene had to be expressed at each time point. Therefore, it may act in some capacity outside the developmental stage in which it is commonly associated. For example, Penfield *et al*. [60] concluded that factors controlling cotyledon expansion in imbibed seeds -- a gibberellin mediated processes -- continue well into seedling establishment. The CHO1 AP2 domain TF that functions in the glucose signaling pathway downstream of ABI4 also appears to function well into seedling establishment [61]. In support of these views, it is interesting that we observe expression of genes known to be involved in the imposition of dormancy well into seedling establishment.

*ABI3 *expression decreases within two days of imbibition to remove dormancy and permit seed germination. ARF10 is believed to increase seed sensitivity to ABA [62] and its delayed suppression would likely contribute to a graded control of ABA responses while *ABI3 *transcript levels decline. In contrast, the levels of *SLR/IAA14 *transcripts are increasing until day 4-5. SLR/IAA14 is known to repress *ARF7 *and *ARF19 *during initiation of lateral roots [63] and it is interesting to speculate that it may play an additional role to restrict the expression of *ARF10 *to vascular tissue in cotyledons and roots in older seedlings [43]. Even if metabolic regulation of highly correlated genes is shown not to occur or is minimal, observing the behavior of expression within a broader physiological and biochemical context through a network correlation analysis may reveal as of yet unknown interactions.

### Identifying mechanisms of metabolic regulation

Imbibition results in seeds undergoing a period of anoxia during which lactate production occurs [1,41]. In animals, elevated lactate has been shown to alter gene expression in certain tumor types [64-66] and may involve carbohydrate response-like elements [67]. We looked for elements within the promoters of photosynthetic genes correlated to lactate and unkM1.85 as a start toward identifying regulatory mechanisms (Supplemental Table 2) in a manner analogous to co-regulated genes identified by microarray analysis [68]. Genes correlated with lactate contained Ocs-like elements responsive to oxidative stress, auxin and salicylic acid [69,70] and motifs showing similarity to those involved in light-responses. Since it is difficult to distinguish between the effects of light and metabolic stimulus [71,72], it is possible that elements identified as light responsive might be metabolite responsive instead. Interestingly, the potential promoter motifs identified in the photosynthetic genes correlated with metabolite unkM1.85 predominantly included elements associated with response to various stresses and did not contain any light-responsive elements. The differences between the potential promoter motifs identified in the two sets of photosynthetic genes indicate that distinct regulatory mechanisms may be operate in groups of genes that may be considered initially as functionally similar through ontological classification. Identification of the TFs that bind to these motifs, and the characterization of identified, but unknown promoter elements will help elucidate the signaling pathways involved in the expression of these genes and potential involvement of metabolic regulation.

The pair-wise analysis of metabolite and transcript levels appears to be a useful investigatory tool to identify potential links between genes and metabolites, thereby providing a number of targets for further examination. However, the identification of a correlated gene and metabolite does not provide information relating to the causality within the relationship, *i.e*. metabolite affecting gene expression or *vice versa*. It is also difficult to determine whether the observed relationship results from a direct interaction between a gene and a metabolite, or whether a downstream signaling event is involved. Such questions can be addressed, in part, by repeating the metabolite measurements in the appropriate mutant and/or by direct measurement of transcript levels in rigorously controlled metabolite feeding experiments.

## Conclusions

A systems biology approach was adopted to investigate the interactions of metabolites and gene expression during seedling development. Both transcript and metabolite data were analysed at various levels and the results visualized using PCA and correlation-based network cartography. The analysis of transcript data alone showed that germination and seedling development is marked by stages of differing gene expression activity. These stages fall at important developmental points, such as at the beginning of seedling emergence, the end of reserve mobilization and the onset of leaf formation. Metabolite levels were revealed to fall into two clusters that reflect the pattern and timing of change, principally those that decrease post-imbibition and those that show a transient increase after seedling emergence. Network cartography, whereby the degree of correlation between variables was used as the basis of sample comparison, provided a clearer picture of the relationship among samples than PCA. This network analysis indicates a shift in the state of nutritionally important metabolites precedes the major shift in transcriptional state going from germination to seedling emergence. Therefore, a suitable metabolic state achieved prior to germination may be necessary for the initiation of gene expression programs for efficient seedling development. Some aspects of gene expression may be regulated by specific metabolites. The key is to identify signaling metabolites and the genes they affect, which may be accomplished by holistic profiling and correlation analysis. In addition, network correlation analysis may reveal component interactions when visualised within the context of a dependent or regulatory process, such as we noted with potential TF relationships uncovered by metabolite correlations.

## Methods

### Plant material and growth conditions

All *Arabidopsis thaliana *L. (ecotype Col-0) seeds were surface sterilized and sown onto 0.8% agar media plates containing 1/2-strength MS salts, pH 5.7 [73]. The sowing density was approximately 500 seeds evenly spread on a 9 cm Petri dish. Agar and MS salts were purchased from Fisher Scientific and Sigma, respectively. The edges of each plate were wrapped in 3 MM surgical tape and the plates were incubated in the dark at 4°C for 4 days before transferring to the growth room. Transfer of plates occurred at 09:00 h. Seeds were germinated at 20°C at 70 μmol of photons m^-2 ^s^-1 ^constant illumination using standard white fluorescent bulbs (General Electric). A drop of less 2 μmol of photons m^-2 ^s^-1 ^was observed going from the centre to the edges of the shelf. We used seeds from completely brown siliques that had been after-ripened for at least 3-weeks after harvesting. The seeds were imbibed for 4 days prior to transfer to the growth room with dormancy being maintained by incubation at low temperature. Images of the stages at which we selected seedlings for analysis is given in Rylott *et al*. [39].

### Design of tissue sampling

Sample harvesting and preparation was conducted in three sets of nine samples with each set encompassing the time points Day 0 to Day 8 after transfer to the growth room [39]. We alternated tissue harvesting regimes to obtain sets of tissue for total RNA and metabolite extraction, thus corresponding tissue samples were used to compare metabolite and transcript profiles. A shelf in the growth room was divided into three sections and plates for each set were arranged horizontally around the shelf, such that each section contained an equal number of plates. Tissue was harvested each day at 09:00 h and only one sample per day was harvested resulting in three biological replicates for each time point. Each tissue sample consisted of pooled seedlings from an equal number of plates from each section. The total number of plates selected for each sample varied depending on the developmental stage. Each plate was examined under a microscope to ensure that seedlings were harvested at the required stage of development. Only those plates with greater than 95% of seedlings at the appropriate developmental stage were used. For sample days 0-4, approximately 0.4 g of seeds or seedlings was washed from the surface of the agar petri dishes with distilled sterile water into a filtration unit. Once the water had passed through, the seedlings were washed in 10 ml more water, weighed and immediately frozen in liquid nitrogen. For sample days 5-8, approximately 0.4 to 0.5 g of seedlings were removed from plates by forceps, rinsed briefly, and immediately frozen in liquid nitrogen. The time from opening the petri dish to freezing the sample was at most 3 min with rinsing times for all samples being within one and a half to two minutes.

### Transcript profiling and DE Analysis

Total RNA was isolated using a borate-based extraction protocol [4]. Production of labelled cDNA, quality checking, and slide hybridization were conducted as described in by Armengaud *et al*. [74]. For each labelling reaction 1 μg of Oligo dT_20 _primer was added to 100 μg of total RNA in a total reaction volume of 20.5 μl. Printed 70-mer oligonucleotide microarrays were obtained from the laboratory of Prof. David Galbraith at the University of Arizona. Versions 1 and 3 arrays containing 29 K elements were used in these experiments. The identity of each spot in the meta-grid was obtained from the Galbraith laboratory http://www.ag.arizona.edu/microarray/. Only one cDNA sample was hybridized per slide. Since transcriptome profiles were produced from more than one microarray print version, only those genes common to all microarrays were used in this analysis.

Hybridized slides were scanned using an Affymetrix 428 scanner set on a gain setting to yield no more than 10 saturated spots per slide and gain settings were varied to account for the quality of the hybridization. Spot checking and intensity determination were done using ImaGene™ (BioDiscovery Inc., CA, USA). The quantified gene expression data produced by ImaGene was normalized using GeneSight™ version 4.1 (BioDiscovery Ltd.). Background signals were subtracted and spots designated as poor hybridization events were discounted from future analysis. In order to address the problem of negative spots, signal intensities below a set value of 20 were raised to that value. The raw expression data after spot removal has been deposited into the ArrayExpress database under the accession number M-MEXP-2493 http://www.ebi.ac.uk/microarray-as/ae/. A standard normalization procedure was applied to the quantified gene expression values obtained for each printed microarray to facilitate comparisons among individual microarrays (Affymetrix GeneChip Expression Analysis technical Manual, 2004). In brief, the top and bottom 2% of the signal values were removed and the mean calculated for the 96% of the values remaining. A value, the scaling factor, was calculated to adjust the mean of the remaining values to 100. Each of the signal intensities on the array was then multiplied by the appropriate scaling factor to normalize signal intensities on an array-by-array basis. A file of combined normalized data is also available from ArrayExpress under the accession number E-MEXP-2493. A group of differentially up-regulated and down-regulated genes was identified between each day at a confidence interval of 99% based on the bootstrapping procedure reported by Kerr & Churchill [75], which is resident within Genesight™. The groups of differentially expressed genes were filtered further with a threshold cut-off value of 1.5-fold. Functional analysis by ontology was done using the information obtained from the TAIR database http://www.arabidopsis.org.

### Quantitative RT-PCR

In order to verify the robustness of our hybridizations and data normalization, we compared the relative expression levels of the genes encoding ACT2 (*ACT2*, At3g18780) and ribosomal protein genes S9 (At1g74970) and L32 (At5g46430) from the arrays to expression values obtained by qRT-PCR. The array and RT-PCR values were normalized by the expression levels of ubiquitin 10 (At4g05320) to allow direct comparison of data from the two different quantitative techniques. The levels of transcripts in each extract were determined by real-time RT-PCR according to Love *et al*. [76]. Transcript values for each reference gene were obtained using a standard curve produced from purified plasmid DNA containing the appropriate cDNA. All genes (except UBQ10) were chosen, because each was spotted between 13 and 27 times throughout the microarray depending on the version used, and thus the average intensity would provide a general indication of the quality of the hybridization. These genes were selected for qRT-PCR analysis prior to hybridization and processing of the arrays, therefore they represent an unbiased indication of the quality of the array hybridization. A plot of array *versus *qRT-PCR values produced lines with high correlation (near unity for *ACT2 *and *RPS9*) demonstrating data of suitable quality for subsequent statistical analysis (Additional File 1).

### Metabolite extraction and quantification

Metabolites were extracted according to Weckwerth *et al*. [77] and quantified by ^1^H-NMR as described in Moing *et al*. [78]. Briefly, the dried extracts were resuspended in 400 mM phosphate buffer pH 6.0 in D_2_O and analyzed at 500.162 MHz on a Bruker spectrometer (Bruker Biospin Avance). Special care was taken to allow absolute quantification of the individual metabolites through addition of ethylene diamine tetraacetic acid sodium salt solution (5 mM final concentration in the NMR tube) to improve the resolution and quantification of organic acids such as malate and citrate, adequate choice of the NMR acquisition parameters (pulse angle 90°, relaxation delay 10 s) and use of an electronic reference (ERETIC mode [79]) calibrated with glucose, fructose, glutamine and glutamic acid sodium salt solutions as described previously [78]. Individual metabolites were identified using published data [78,80,81], acquisition of NMR spectra of reference compounds under exact solvent conditions, and spiking extracts with reference compounds. They were quantified using the metabolite mode of AMIX software (Bruker Biospin v. 3.5.6) based on the number of protons comprising the corresponding resonance. Concentrations in the NMR tube were converted to amounts per g fresh weight using the mass of sample extracted. Citrate, formate, fumarate, glutamate, lactate and malate are expressed as μg of the acid form. The concentration of NMR unknown compounds (named according to the form of the resonance, S for singlet, D for doublet, M for multiplet, and its frequency in ppm) was calculated on the assumption that the measured resonance corresponded to one proton and using an arbitrary molecular weight of 100 Da. We verified the robustness of the quantification procedure by observing a near 1 to 1 relationship between levels of metabolites when we compared those measured in a mixture of samples, one from each day, with the corresponding calculated theoretical mix (Additional File 1).

### Data analysis for network cartography

For the network cartography and correlation analysis, only those genes which had at least one expression value for each of the sample days were included in the analysis. This pre-processing step produced a set of 10,005 genes. Similar data pre-processing was not required for metabolite levels as they had been quantified absolutely for each extract. Since material for both the transcript and metabolite profiling was collected in three independent groups, the values were averaged for each day. Hierarchical, *K*-means and 2D-SOM clustering were done using the metabolite data imported into Genesight ™. PCA was performed using MATLAB™ release 2007b ((The MathWorks, Natick, MA). Visualization of significant correlations, i.e. network cartography, was conducted using springScape [37]. Pearson correlation coefficients (signed *r *value) were used to generate the similarity matrices for the spring embedding of metabolite, transcript and combined data. MATLAB™ release 2007a was the mathematics platform for the spring embedding and metabolite-gene correlation analysis. Depending on the data set being analysed, the initial similarity matrix was cut at a threshold value to facilitate the spring embedding and to enhance the significance of the output correlations. For the similarity matrix of correlations between the 27 individual metabolites, a Bonferroni adjustment was applied based on a significance value of 0.1 and 351 tests. The day-by-metabolite and the day-by-gene similarity matrices were adjusted strictly by correlation coefficient in order to compare directly clustering between the two data sets. A FDR [42] was used to threshold the level of significance for the metabolite by gene correlations based on a significance value of 0.1 and 276,345 (27 × 10,235) tests. Determination of metabolites differentially expressed between the two groups of days was conducted by Student's t-tests also using a FDR of 0.1 (6 × 20 tests) to determine significance.

## Abbreviations

ABA: abscisic acid; DE: differential expression or differentially expressed; DR: down-regulated (regulation); FDR: false discovery rate; FW: fresh weight; GA: gibberellic acid; PCA: principal component analysis; qRT-PCR: quantitative real-time PCR; SOM: self-organising map; TF: transcription factor: UR: up-regulated (regulation)

## Authors' contributions

EA contributed to experimental design, prepared and extracted all tissue samples, processed microarray data and contributed to manuscript preparation. AM quantified NMR data and contributed to manuscript preparation. TMDE did the network analysis and helped write the manuscript. MM collected and pre-processed NMR data. ADT aided in experimental design and contributed to manuscript preparation. DR contributed to experimental design and manuscript preparation. MAH was principal investigator and experimental designer, did the PCA analysis and was the major editor of the manuscript. All authors have read and approved the final manuscript.

## Supplementary Material

Additional file 1 **Supplementary Data.** Supplemental Figure 1. Levels of metabolites over the developmental series; Supplemental Figure 2: PCA of variation among days based on transcript levels. Supplemental Figure 3: PCA of variation among days based on metabolite levels; Supplemental Figure 4: Scatter plots showing individual correlations between lactate and transcripts. Supplemental Figure 5: Comparison of microarray and qRT-PCR expression data; Supplemental Figure 6: Comparison of calculated and measured metabolite levels in mixtures of tissue extracts. Supplemental Table 1: Significant metabolite changes between groups of days. Supplemental Table 2: Motifs found in promoter sequences of photosynthetic genes correlated with lactate. Click here for file

Additional file 2 Filtered Gene List. Click here for file

Additional file 3 List of genes correlated with metabolites. Click here for file
