# Supplementary material for: Correlation Network Analysis reveals a sequential reorganization of metabolic and transcriptional states during germination and gene-metabolite relationships in developing seedlings of Arabidopsis
Source: BMC Syst Biol. 2010 May 13;4:62. doi: 10.1186/1752-0509-4-62 (PMC2890501; doi:10.1186/1752-0509-4-62)
Supplement: Additional file 1 — Supplementary Data. Supplemental Figure 1. Levels of metabolites over the developmental series; Supplemental Figure 2: PCA of variation among days based on transcript levels. Supplemental Figure 3: PCA of variation among days based on metabolite levels; Supplemental Figure 4: Scatter plots showing individual correlations between lactate and transcripts. Supplemental Figure 5: Comparison of microarray and qRT-PCR expression data; Supplemental Figure 6: Comparison of calculated and measured metabolite levels in mixtures of tissue extracts. Supplemental Table 1: Significant metabolite changes between groups of days. Supplemental Table 2: Motifs found in promoter sequences of photosynthetic genes correlated with lactate. [file 1752-0509-4-62-S1.PDF]

Supplemental Material

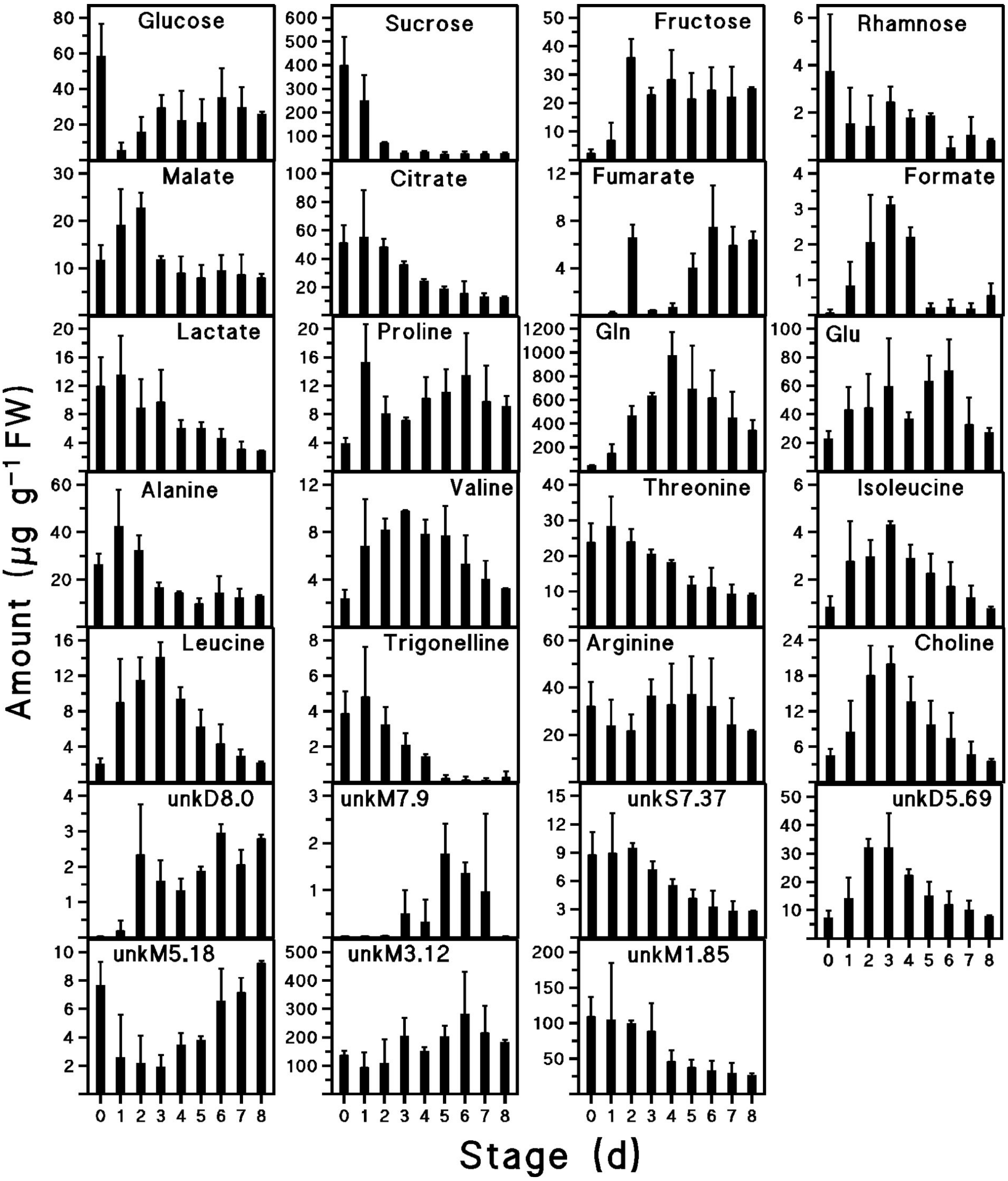

Supplemental Figure 1. Levels of various metabolites over the developmental series. Levels of metabolites were determined in tissue extracts by ^{1}H-NMR according to Moing et al. (2004). The letters and numbers of unknown peaks represent the type of signal and the chemical shift relative to trimethylsilyl propionic acid-d4 sodium salt (TSP), the internal and chemical shift standard: D, doublet; M, multiplet; S, singlet. Values represent the average ± sd of three independent tissue samples, except for day 3, which is the average ± range of two samples.

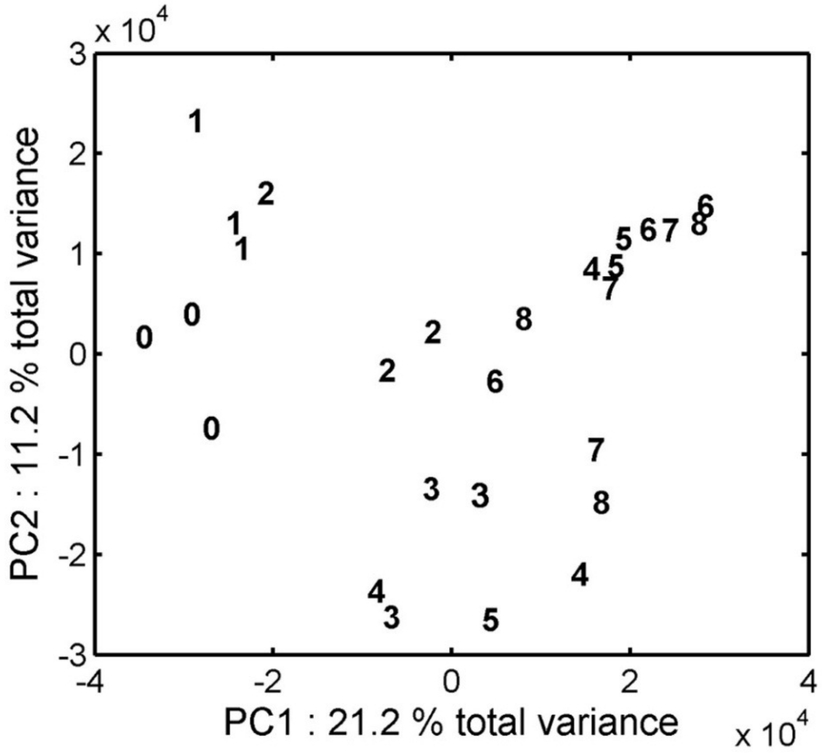

Supplemental Figure 2. Principal component analysis of the time points sampled during germination and seedling establishment based on the transcript levels of all 27 arrays. Each number 0 to 8 represents one day (24 h) from imbibed seeds (0) to 8 days (8) of age, respectively.

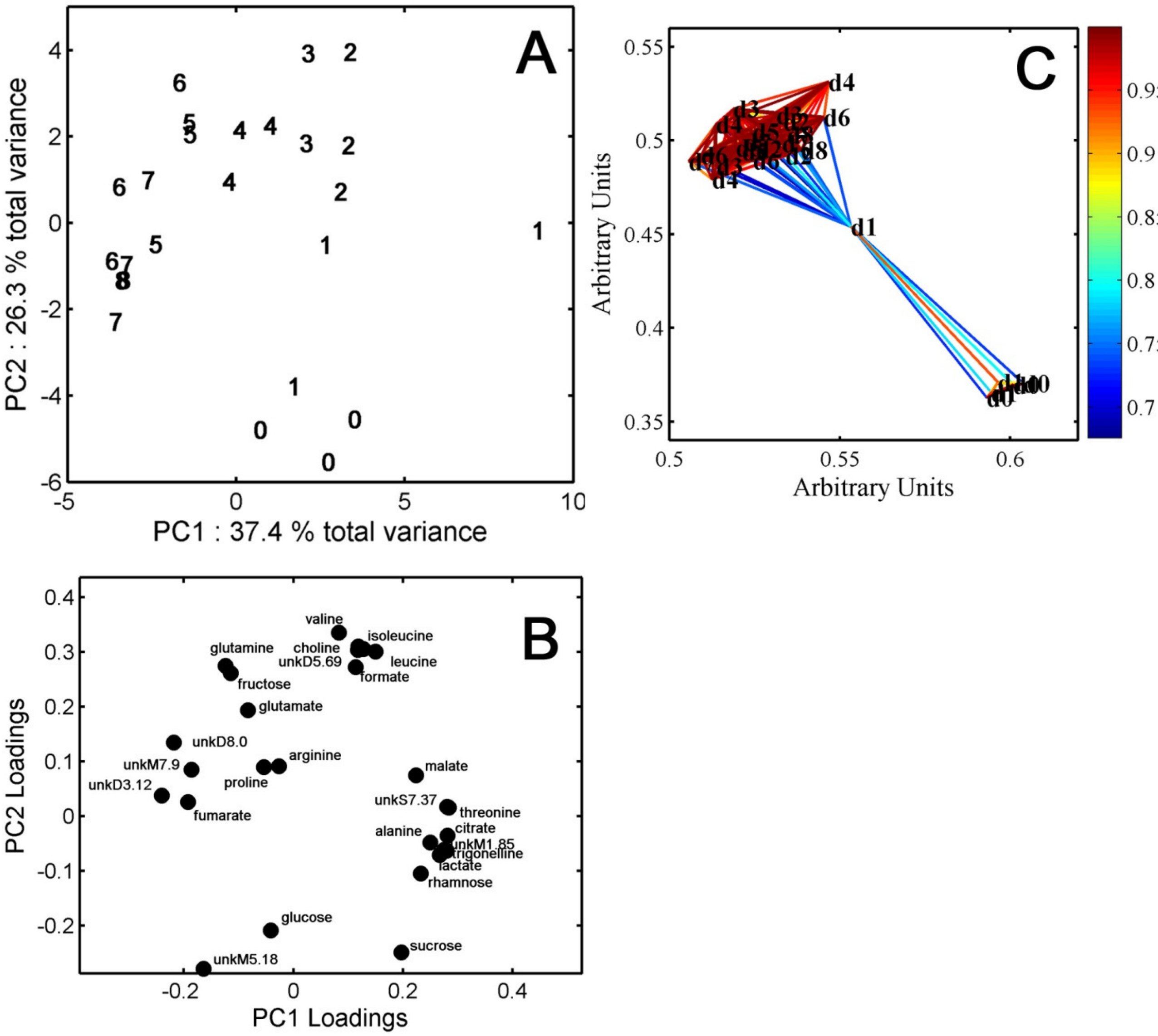

Supplemental Figure 3. Principal component analysis of metabolite levels where each number represents one day (24 h) from imbibed seeds (0) to 8 days of age (8). A, PCA scores plot based on metabolite levels for each sample day, except for day 3 where only 2 samples are included. B, Loadings plot for mean-derived PCA scores plot shown in B. C, springScape plot of individual days based on metabolite levels.

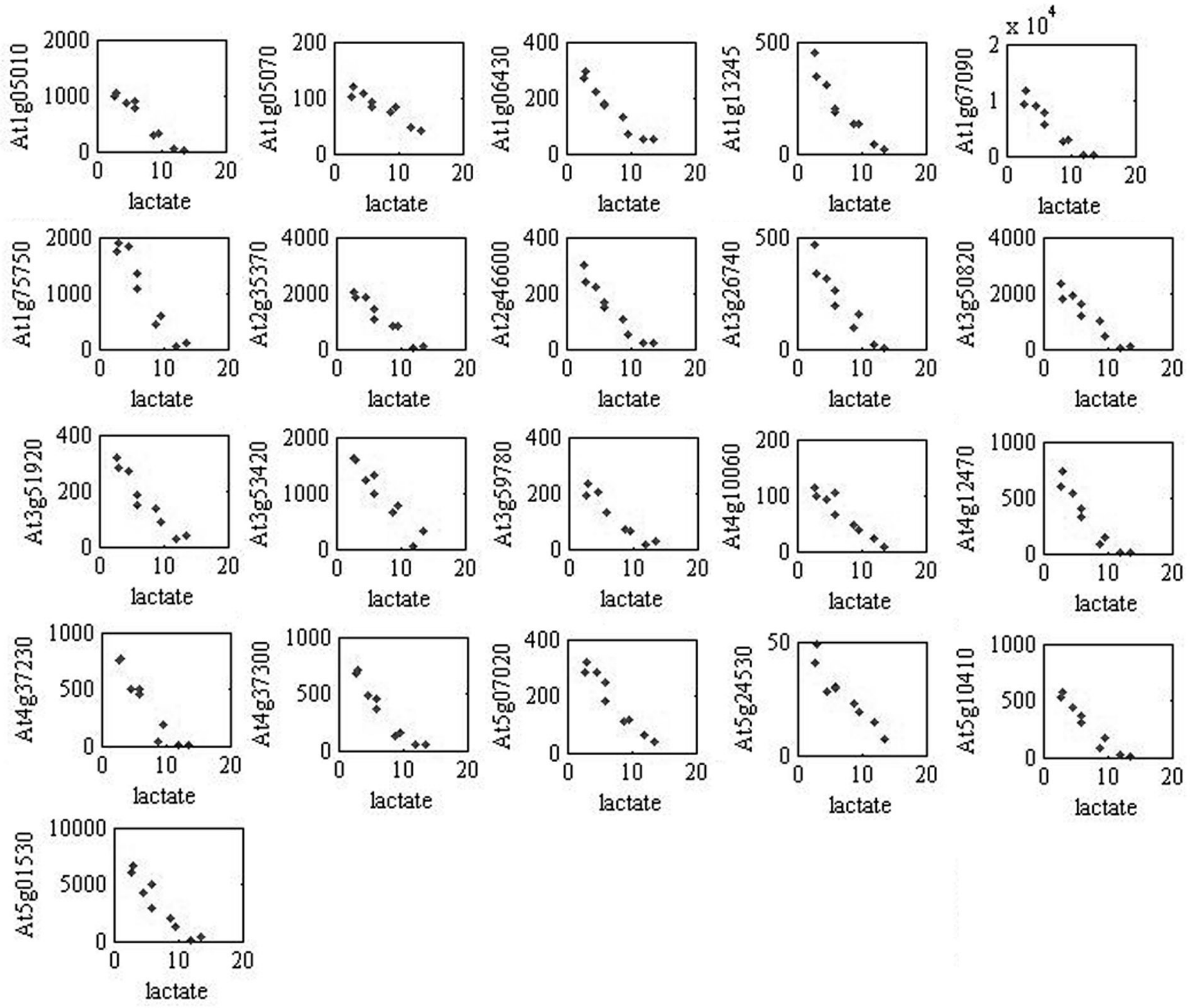

Supplemental Figure 4. Scatter plots showing the relationships between lactate levels and transcript levels for highly correlated genes. Pearson correlation coefficients were calculated between lactate and transcript levels for every gene in the dataset. The scatter plots are shown only for correlations with $p < 0.0001$ for an n-1 value = 8.

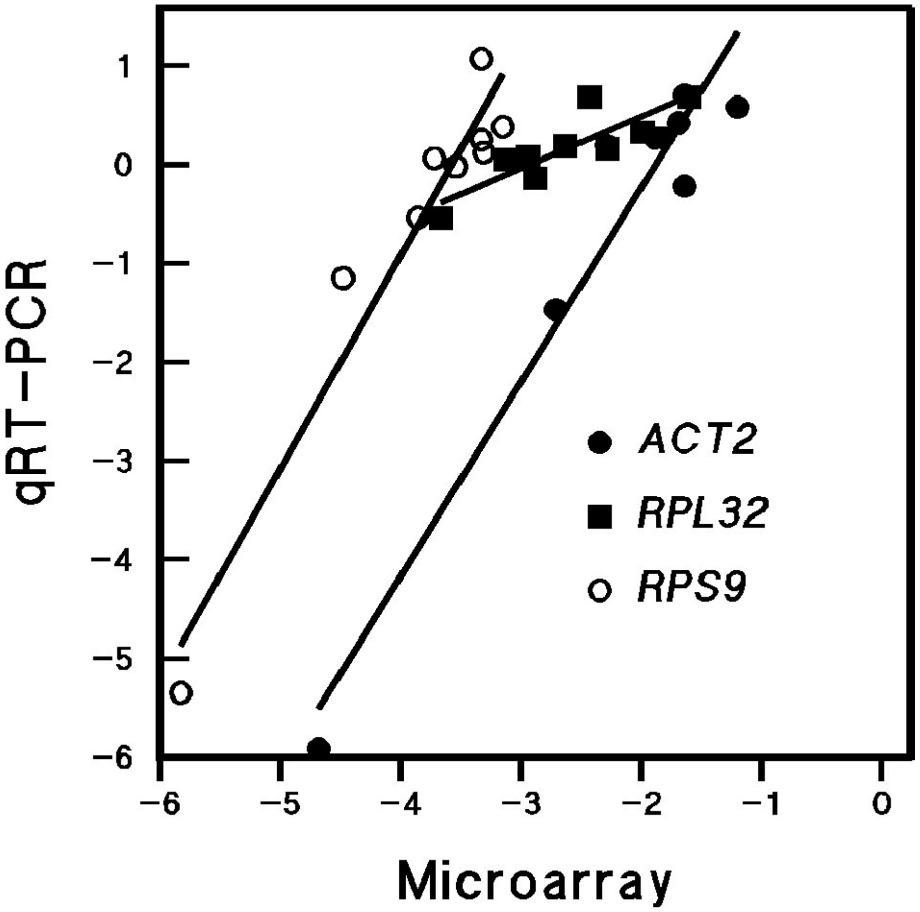

Supplemental Figure. 5. Comparison of selected microarray and qRT-PCR expression data. *ACT2*, *RPL32* and *RPS9*, were chosen as controls, since they were spotted multiple times on each version of the Arizona arrays. Ubiquitin was spotted only once, but the spots were checked visually on each array for quality. On only two of 27 slides the ubiquitin spot was not of sufficient quality to be included in the analysis. The intensity value expression of the reference genes *ACT2*, *RPL32* and *RPS9* were normalized to the expression of Ubiquitin for both the microarray and the qRT-PCR expression values. The ratios were log-transformed (base 2) to permit visualization of trends. Each point represents the average value of three measurements for each day (error bars are not shown for clarity, but standard error for each average value was less than 10%), except for *RPL32* day 0 qRT-PCR, which was one value. The R^2 values for *ACT2*, *RPL32* and *RPS9* were 0.9322 (p = 2.4x10^{-5}), 0.7272 (p = 0.0035), and 0.9338 (p = 2.3x10^{-5}), respectively.

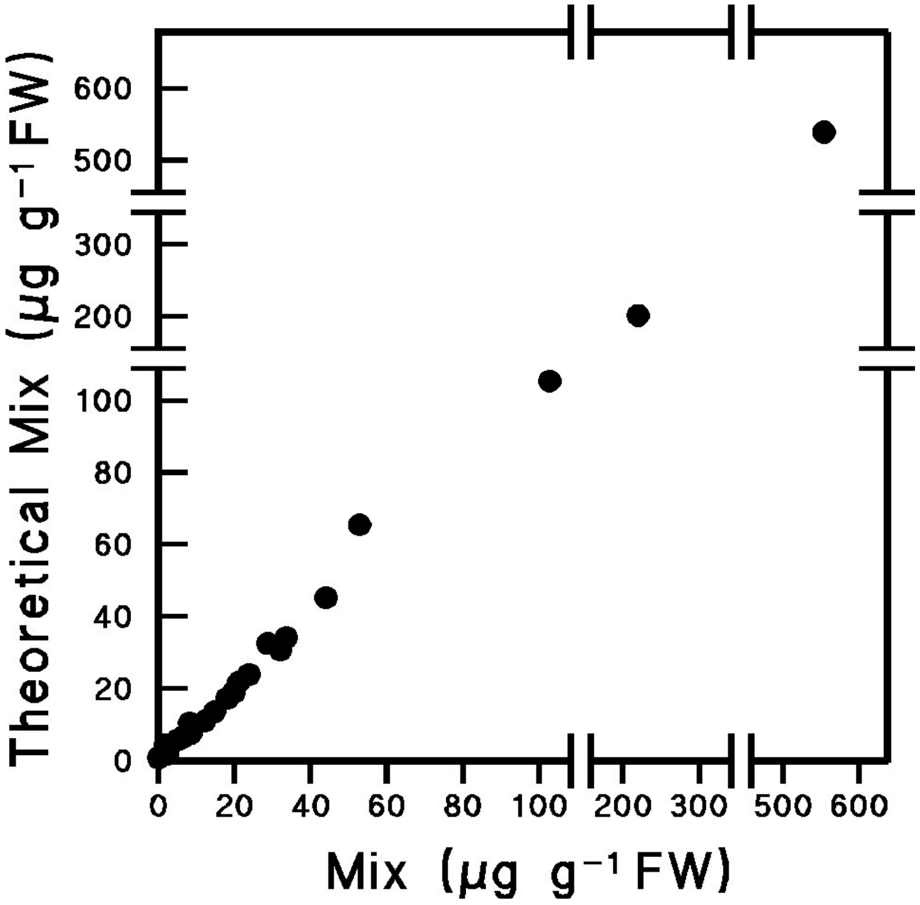

Supplemental Figure. 6. Comparison of calculated and measured metabolite levels in mixtures of tissue extracts. One sample from each day was selected at random and equal volumes of the 9 samples were combined to produce a mixed sample for which the levels of each metabolite were determined by ^{1}H-NMR. The theoretical level of each metabolite was calculated from the values from the individual samples. The regression value $r^2 = 0.9999$ with $p < 0.0001$.

Supplemental Table 1. Metabolites changing between days 0-1 and days 2-8. An FDR = 0.1 ($P < 0.0304$) was used to establish significance for 6 x 20 tests.

Metabolite	T value[a]	P value
Alanine	2.9696	0.0067
Arginine	-0.589	0.5614
Choline	-1.5384	0.137
Citrate	2.3279	0.0287
Formate-	1.4298	0.1657
Fructose	-3.2303	0.0036
Fumarate	-2.3008	0.0304
Glucose	0.1161	0.9086
Glutamate	-1.5592	0.132
Glutamine	-3.6281	0.0013
Isoleucine	-0.9497	0.3517
Lactate	4.0947	0.0004
Leucine	-0.9598	0.3467
Malate	0.5964	0.5565
Proline	-0.4686	0.6436
Rhamnose	3.1019	0.0049
Sucrose	9.5738	0
Threonine	2.0873	0.0477
Trigonelline	3.8902	0.0007
valine	-1.4586	0.1576
unkD3.12	-1.5032	0.1458
unkD5.69	-1.8561	0.0758
unkD8.0	-6.1614	0
unkM1.85	3.089	0.005
unkQ5.18	-0.3216	0.7505
unkQ7.9	-1.6744	0.107
unkS7.37	1.4888	0.1496

[a] Positive T shows higher in the days 0 and 1 group.

Supplemental Table 2. Motifs found in promoter sequences of photosynthetic genes correlated with lactate.

Motif Submitted[a]	Sequence Returned[a]	Descriptor
TTGRATTG	No match	
GCCACN	ACGTGGC	ACGT-containing ABA response element
	ACATAAT AGCCAC ATATT	Box II – light responsive element
	GCCACT	CAT box – meristem Specific expression
	TCCTCGT GGCA	G-box – light responsive element
	CATCGTG TGGC	GTGGC motif -- light responsive Element
	AGCCACC	Box S – wound and pathogen response
	GCCACN	light responsive Element
CAGACA	No match	
TCAAT	ATCTTAT GTCATTG ATGACGA CCTCC	OCS element -- induced in auxin, oxidativestress and salicyclic acid responses

[a]Where nucleotide ambiguity occurs, the IUPAC nucleotide symbol system has been used.
